# E-test versus agar dilution for antibiotic susceptibility testing of *Helicobacter pylori*: a comparison study

**DOI:** 10.1186/s13104-019-4877-9

**Published:** 2020-01-10

**Authors:** Muhammad Miftahussurur, Kartika Afrida Fauzia, Iswan Abbas Nusi, Poernomo Boedi Setiawan, Ari Fahrial Syam, Langgeng Agung Waskito, Dalla Doohan, Neneng Ratnasari, Ali Khomsan, I. Ketut Adnyana, Junko Akada, Yoshio Yamaoka

**Affiliations:** 1 Division of Gastroentero-Hepatology, Department of Internal Medicine, Faculty of Medicine-Dr. Soetomo Teaching Hospital, Universitas Airlangga, Jalan Mayjend Prof. Dr. Moestopo No. 6-8 Surabaya, Surabaya, 60131 Indonesia; 2 Institute of Tropical Disease, Universitas Airlangga, Surabaya, 60115 Indonesia; 3 Department of Environmental and Preventive Medicine, Oita University Faculty of Medicine, Yufu, Oita 879-5593 Japan; 4 Division of Gastroenterology, Department of Internal Medicine, Faculty of Medicine, University of Indonesia, Jakarta, 10430 Indonesia; 5 Department of Internal Medicine, Faculty of Medicine Gadjah, Mada University-Dr. Sardjito, Yogyakarta, 55281 Indonesia; 6 Department of Community Nutrition, Bogor Agriculture University, Bogor, 16680 Indonesia; 7 School of Pharmacy, Bandung Institute of Technology, Bandung, 40132 Indonesia; 8 Global Oita Medical Advanced Research Center for Health, Oita University, Oita, 870-1192 Japan; 9 Department of Medicine, Gastroenterology and Hepatology Section, Baylor College of Medicine, Houston, TX 77030 USA

**Keywords:** E-test, Agar dilution, *Helicobacter pylori*, Antibiotic susceptibility test

## Abstract

**Objective:**

For evaluating the antibiotic resistance of *Helicobacter pylori*, the agar dilution method is the gold standard; however, using this method in daily practice is laborious. E-test has been proposed to be an uncomplicated method. This study was aimed at validating the E-test and detecting the presence of any bias between the agar dilution method and E-test.

**Results:**

The agar dilution method and E-test were performed using five antibiotics for 72 strains of *H. pylori* obtained from clinical patients in Indonesia. The E-test’s results showed a higher prevalence of resistance to all the antibiotics tested but the difference was not significant. Results showed high essential agreement (> 90.0%) for all the antibiotics, but only 84.7% for metronidazole. The agreement for MIC value was acceptable for levofloxacin, clarithromycin, and metronidazole. For amoxicillin, it showed only fair agreement (0.25) by the Kappa analysis and significant difference by Passing-Bablok regression. Even though some discrepancies were found, the E-test has an acceptable agreement for levofloxacin, metronidazole, tetracycline, and clarithromycin but further confirmation may be necessary for amoxicillin.

## Introduction

*Helicobacter pylori* virulent strains being predominant in several regions thus *H. pylori* should always be treated regardless of the presence of symptoms [1]. However, antibiotic resistance caused in *Helicobacter pylori* eradication failure and the resistance rates widely vary around the world [2–4]. Recent hospital-based study in Japan reporting 48% of clarithromycin resistance [5] while our previous study in Indonesia reported a 9% resistant rate [6]. Because of such differences, the Maastricht Consensus Report on *H. pylori* infection has stated that antibiotic susceptibility tests with periodic monitoring should be performed in each region to determine the most suitable therapy for a given population [2].

Given the importance of antibiotic susceptibility testing for *H. pylori*, it is crucial to choose a testing method that delivers high accuracy and feasible for clinical settings. According to the Clinical and Laboratory Standards Institute (CLSI), direct measurement with the agar dilution method (ADM) is the gold standard for *H. pylori* [7]. However, ADM requires laborious preparation and may not cost and time-effective for daily clinical practices [8]. An alternative, relatively simple method is the E-test, which uses different concentrations of antibiotics in a single strip.

In the present study, we followed CLSI guideline EP-09 to compare the measurement procedures and estimate bias [9]. These methods have been applied in other studies, but rarely for *H. pylori* [10–13]. Thus, a study using isolates from the Indonesian population, an area with the variable prevalence and virulence type of *H. pylori* [6, 14] may provide another insight of the E-test reliability.

This study aimed to examine the susceptibility of Indonesian *H. pylori* isolates to five antibiotics (amoxicillin, clarithromycin, metronidazole, tetracycline, and levofloxacin), using ADM as the gold standard to validate the level of agreement and reliability of the E-test.

## Main text

### Methods

#### Patients and H. pylori isolates

We analyzed a total of 72 clinical *H. pylori* isolates obtained from adult dyspeptic patients as part of a nationwide survey in Indonesia and reference strain ATCC26695. Data for the E-test results for the 72 strains were published in our previous study [6]. Isolate storage and all the susceptibility tests were conducted at the Department of Environment and Preventive Medicine, Oita University Faculty of Medicine, Yufu, Japan.

All the patients from whom the isolates were obtained provided written informed consent, and the study protocol was approved by the Institutional Review Board or Ethics Committee of Dr. Cipto Mangunkusumo Teaching Hospital (Jakarta, Indonesia), Dr. Soetomo Teaching Hospital (Surabaya, Indonesia), Dr. Wahidin Sudirohusodo Teaching Hospital (Makassar, Indonesia), and Oita University Faculty of Medicine (Yufu, Japan).

#### Antibiotic susceptibility testing by ADM and E-test

The procedure for ADM followed the protocol described by CLSI [7, 15]. Briefly, around 2 µL of bacterial suspension with 0.5 McFarland concentration was inoculated into Mueller–Hinton agar contained twofold dilutions of antibiotics. E-test (bióMeurieux, La Balme-Les-Grottes, France) procedure was reported previously, following manufacturer instructions [6]. Briefly, 100 µL of *H. pylori* suspension with 3 McFarland standard was inoculated into Mueller–Hinton agar plate without antibiotic and one E-test strip were applied to the center of the plate. incubation under microaerophilic conditions for 72 h. A full explanation of the method is available in the Additional file 1: Fig. S1.

#### Statistical analysis

The ADM is the gold standard for antibiotic susceptibility test, thus the results were used as the reference for validating the E-test method. The samples were grouped into “sensitive” and “resistant” according to the EUCAST clinical breakpoint criteria [16] and were then used to evaluate, essential agreement, Cohen’s kappa analysis, McNemar, also the major and very major error rate between the two methods. The analysis was performed using the SPSS statistical software version 23.0 (IBM Corp., Armonk, NY, USA).

To better understand the agreement of the MIC results, we followed CLSI guideline EP-09 to use the non-parametric approach proposed by Bland and Altman with Krouwer modification because the gold standard is available [17, 18]. Scatter plot and Passing–Bablok analyses were also performed using R environment ver. 3.5.1 with the mcr package [19]. Receiver operating characteristic analysis was used to evaluate the sensitivity, specificity, and area under the curve (AUC) of the E-test results relative to those of the ADM.

### Results

#### Susceptibility results of the ADM and E-test

We initially attempted to culture 77 strains of *H. pylori*; however, five strains did not grow and were excluded. This growth failure number was still acceptable according to the U.S. Food and Drug Administration (FDA) recommendation for antimicrobial susceptibility testing [20]. Both the ADM and E-test were applied to the same 72 remaining strains.

In Table 1, both ADM and the E-test showed high resistance rates to metronidazole and levofloxacin. Slightly higher resistant rates were shown by the E-test for four antibiotics, but this difference was not statistically significant (McNemar test: amoxicillin, *P* = 0.99; metronidazole, *P *= 0.55; clarithromycin, *P* = 0.63; and levofloxacin, *P* = 0.99; the value for tetracycline could not be calculated because of the zero percentage result). MIC50 and MIC90 were the same for all the antibiotics except amoxicillin and tetracycline.

**Table 1:** Resistance rates according to the agar dilution method (ADM) and E-test

Antibiotic	Clinical breakpoint (mg/L)	Resistance rate (%) n = 72		MIC50^a^ (mg/L)		MIC90^a^ (mg/L)		Essential agreement (%)	Kappa coefficient	95% confidence interval
		ADM	E-test	ADM	E-test	ADM	E-test			
Amoxicillin	> 0.125	3/72 (4.2)	4/72 (5.6)	0.032	0.016	0.064	0.125	93.1	0.25	− 0.38 to 0.88
Metronidazole	> 8	30/72 (41.7)	33/72 (45.8)	8	8	64	64	84.7	0.69	0.52 to 0.85
Clarithromycin	> 0.5	3/72 (4.2)	5/72 (6.9)	0.032	0.032	0.25	0.25	94.4	0.47	− 0.29 to 0.97
Levofloxacin	> 1	18/72 (25.0)	19/71 (26.4)	0.25	0.25	8	8	93.1	0.81	0.66 to 0.97
Tetracycline	> 1	0/72 (0.0)	2/71 (2.8)	0.125	0.064	0.5	0.25	97.2	–	–

^a^MIC50/MIC90: minimum inhibitory concentrations that inhibited 50 and 90% of the isolates. For comparability with ADM, the upper limits were set as 1 mg/L for amoxicillin, 2 mg/L for clarithromycin and tetracycline, 8 mg/L for levofloxacin, and 64 mg/L for metronidazole, and the lower limits were set as 0.016 mg/L for amoxicillin, 0.032 mg/L for clarithromycin and tetracycline, 0.125 mg/L for levofloxacin, and 1 mg/L for metronidazole

#### Agreement of the susceptibility results

To determine the precision and reliability of the E-test. Differences in the “sensitive” and “resistant” interpretations were further analyzed according to the agreement percentage and Cohen’s kappa analysis (Table 1). The kappa values showed fair agreement for amoxicillin, moderate for clarithromycin, and substantial agreement for metronidazole and levofloxacin prevalence by ADM. The kappa value for tetracycline could not be evaluated because of the zero value for prevalence.

#### Measurement of errors

**Fig. 1:**
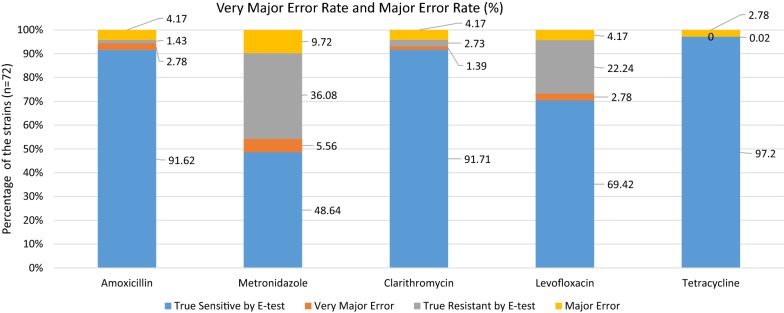
The major error and very major error rates in categorical agreement. A very major error was defined as when a strain that was resistant according to ADM was assessed as “sensitive” by the E-test; this may result in the patient not receiving effective antibiotics, allowing the infection to continue. A major error was defined as when the strain was sensitive according to ADM but assessed as “resistant” by the E-test. True sensitive is sensitive percentage minus very major error rate while the true resistant is resistant percentage minus major error rate

Figure 1 shows the major errors and very major errors, as described in previous studies [13, 21, 22]. For all the antibiotics, the very major error rate was lower than the major error rate. Despite its low resistance rate according to both the ADM and E-test (4.2 and 5.6%, respectively), amoxicillin was associated with a very major error rate of 2.78% and a major error rate of 4.17%. Metronidazole also had a high very major error and major error rates (5.56 and 9.72%, respectively).

#### Analysis of agreement and systematic bias

**Fig. 2:**
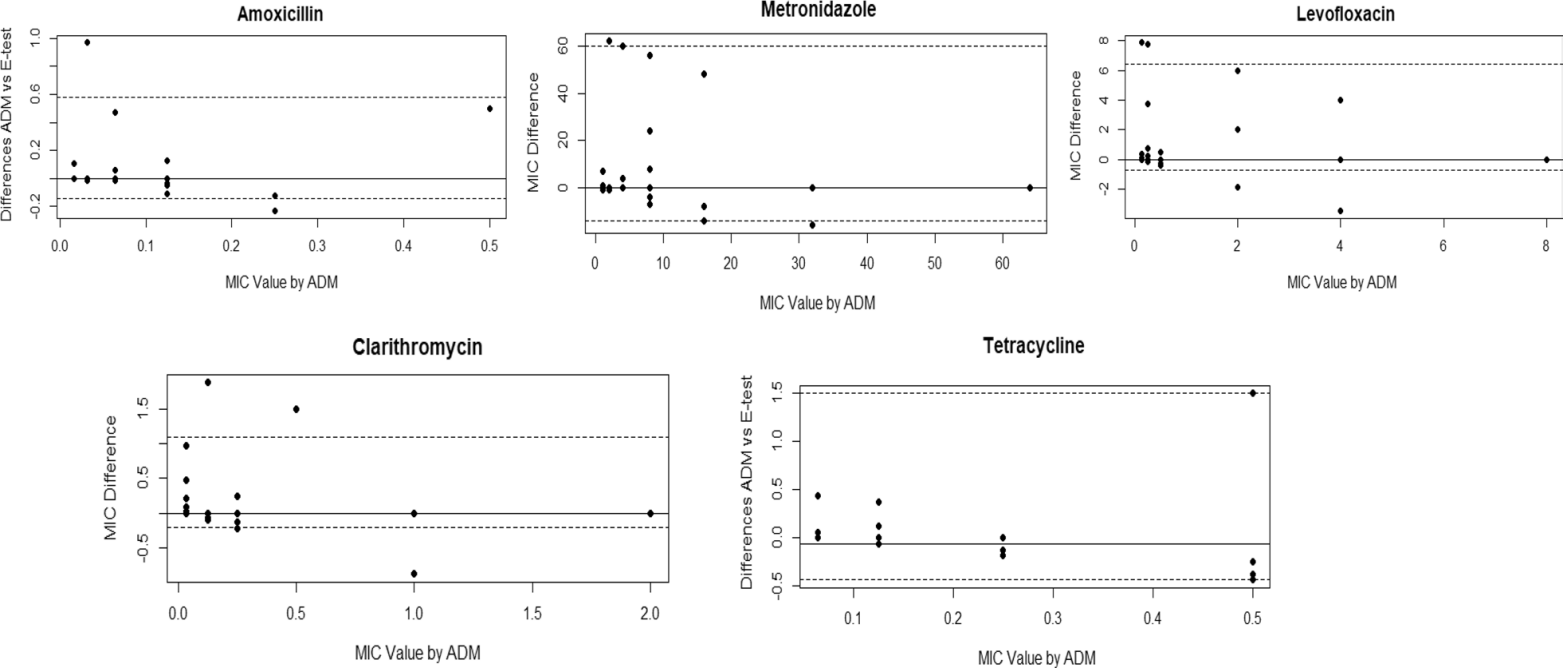
Bland–Altman difference plots comparing the minimum inhibitory concentration (MIC) results of the agar dilution method and E-test for the five antibiotics. The plots use the modification of Krouwer [17, 18]. The plots confirm that the measurements by both methods were equal for all the antibiotics, except for tetracycline. For tetracycline, the median difference was 0.061 mg/L

The modified Bland–Altman difference plots [17, 18] to assess the limit of agreement and any pattern of bias are shown in Fig. 2. For all the antibiotics except tetracycline, the median difference in measured MIC was zero, indicating there was no constant bias between the E-test and ADM. At low MIC values, the differences in the plots tended to clump around the median value, indicating precision between the methods. The error tended to increase at higher values of MIC for all the antibiotics, shown by the greater difference values. Spearman correlation analysis confirmed this correlation was significant for amoxicillin (r = 0.53, *P* ≤ 0.001), clarithromycin (r = 0.56, *P* ≤ 0.001), and tetracycline (r = 0.76, *P* ≤ 0.001).

As recommended by the CLSI, Passing–Bablok regression analysis was used to estimate the analytical method agreement and detecting the presence of any bias. It can determine whether the E-test procedure and agar dilution method can be used interchangeably. In the Passing–Bablok regression plots (Additional file 1: Fig. S1), the confidence intervals for the intercepts for all the antibiotics except amoxicillin and tetracycline included zero, indicating there were no significant proportional differences between the values measured by the two methods. The confidence intervals for the slopes included the value 1 (indicating no significant difference between the measurements) for all the antibiotics except amoxicillin and tetracycline.

#### Accuracy of the E-test

The sensitivity, specificity, and AUC of the E-test for distinguishing the strains resistant to each antibiotic were calculated. For amoxicillin resistance, the E-test showed low sensitivity but high specificity (33.3 and 95.7%), with a good AUC value (0.783). For clarithromycin, the sensitivity was also low (66.7%) but the specificity and AUC were high (95.7 and 0.937%, respectively). For metronidazole and levofloxacin, the E-test showed quite high sensitivity (86.7 and 88.9%, respectively), specificity (83.3 and 94.4%), and AUCs (0.887 and 0.919). It was not possible to make these calculations for tetracycline because ADM showed the resistance rate was zero.

## Discussion

This is the first study to evaluate the validity of the E-test as an alternative method for the detection of resistant strains of *H. pylori* in Indonesia. E-test is preferable in clinical practice due to the lower cost and less time consuming [8]. Several previous studies have validated the E-test for European populations [21–23], Brazilian children [13] and American [24].

In this study, we found that the resistance rate obtained from E-test showed a slightly higher discrepancy compared to ADM, although this was statistically not significant according to McNemar analysis; similar to previous studies [23, 24]. For levofloxacin, metronidazole, and clarithromycin, the essential agreement was in the acceptable range according to the FDA (> 90%) with low major error and very major error rates [20, 25]. Agreement for MIC values with Bland–Altman and Passing-Bablok also showed that both methods can be used interchangeably with good sensitivity and specificity [26, 27].

However, consistent with other studies, metronidazole showed a low essential agreement and high very major error rate, but metronidazole resistance is common and is not a first-line treatment so its use is limited [2, 6, 22, 28]. The emergence of heteroresistance to metronidazole within a mixed colony may have played an important role in this result [24]. For amoxicillin, Cohen’s kappa analysis only showed a fair agreement. Moreover, a non-significant correlation for Passing-Bablok and very low sensitivity might indicate the lower reliability of the E-test for measuring amoxicillin resistance, compared to other antibiotics. However, it also probably due to a very low prevalence of amoxicillin resistant thus limit the kappa analysis in estimating values, known as the “kappa paradox” [29]. Hence, amoxicillin is included in the first line regiment and the non-concordance that occurred may affect the treatment choice and patient’s outcome [2].

The reason for the discrepancy observed in the E-test results was still unknown but is probably the result of several factors. The procedure for storing E-test strips may affect the drug concentration, and incubation in a microaerophilic environment may influence the activity of the antibiotic, especially for macrolides [8, 23]. The large bacterial inoculum size was tested by an E-test strip while in ADM, small number of bacteria were inoculated on agar plates containing antibiotics. The difference in bacteria/antibiotics ratio may have resulted in higher growth capabilities of the strain, resulting in a higher measured resistance rate in the E-test than ADM. Indeed, it has previously been demonstrated that inoculum concentration has an impact on the discrepancies observed in microdilution and ADM [30].

In general, the E-test may overestimate the rates of resistance to antibiotics, but it may be applicable because it showed good agreement with ADM results for levofloxacin, metronidazole, clarithromycin, and tetracycline. However, the disagreement from amoxicillin may require further confirmation.

## Limitations

One of the limitation of the present study was that no tests were performed in other laboratory centers to check the reproducibility of the methods. The isolates used in this study were collected in 2015 and stored in − 80 °C. Even though the freeze storage effect on antibiotic sensitivity in *H. pylori* was varied between the studies [31–33], it may explain the discrepancy of MIC found in this study. The zero resistance rate for tetracycline also affected the analysis. The number of study samples met the recommendation of CLSI; however, a greater number may have improved the precision of the comparison. Nevertheless, the findings of this study provide the data that E-test was a simple but reliable diagnostic tool for antibiotic-resistant detection in *H. pylori.*

## Supplementary information

**Additional file 1.** Methods explanation. **Figure S1.** Passing–Bablok regression of ADM and E-test.

## Data Availability

The data sets used and/or analyzed during the current study available from the corresponding author on reasonable request.

## Abbreviations

ADM: agar dilution method
AUC: area under the curve
CLSI: Clinical and Laboratory Standards Institute
EUCAST: European Committee on Antibiotic Susceptibility Testing
FDA: U.S. Food and Drug Administration
MIC: minimum inhibitory concentration
