# Supplementary material for: E-test versus agar dilution for antibiotic susceptibility testing of Helicobacter pylori: a comparison study
Source: BMC Res Notes. 2020 Jan 10;13:22. doi: 10.1186/s13104-019-4877-9 (PMC6954499; doi:10.1186/s13104-019-4877-9)
Supplement: Supplementary file 1 — Additional file 1. Methods explanation. Figure S1. Passing–Bablok regression of ADM and E-test. [file 13104_2019_4877_MOESM1_ESM.docx]

**Additional File 1. Method**s **Explanation**

The storage and culture methods

The *H. pylori* isolates were stored at −80 °C in Brucella broth (Becton Dickinson, Franklin Lakes, NJ, USA) supplemented with 10% horse serum and 10% glycerol. The isolates were recovered by sub-culturing in the Brucella agar medium (Becton Dickinson) supplemented with 7% defibrinated horse blood in microaerophilic conditions (10% O_2_, 5% CO_2_, and 85% N_2_) at 37 °C, and used for the susceptibility tests.

Agar Dilution Method

The isolates from the frozen stock were sub-cultured twice and collected in a bacterial suspension adjusted to McFarland 0.5. Around 2 µL of bacterial suspension was inoculated into Mueller–Hinton agar supplemented with 5% horse blood and two-fold dilutions of the antibiotics amoxicillin, clarithromycin, metronidazole, levofloxacin, and tetracycline (Sigma Aldrich, St. Louis, MO, US) and incubated 72 h in the microaerophilic environment. The determination of susceptibility was based on the following clinical breakpoints reported by EUCAST [[1](#_ENREF_1)].

E-test Method

Briefly, 100 µL of *H. pylori* that was diluted into a Brucella broth medium and adjusted to 3 McFarland standard on a 90-mm Mueller–Hinton agar plate supplemented with 5% horse blood. After drying for 10 min, one E-test strip (bióMeurieux, La Balme-Les-Grottes, France) was applied to the center of the plate and incubated under microaerophilic conditions for 72 h. The number read on the E-test at the border of the colony growth was recorded as the minimum inhibitory concentration (MIC). However, the range of MIC values from the E-test differed from those from the standardized range of ADM, so the E-test values were rounded up to the next upper two-fold value.

Statistical analysis

According to the CLSI, ADM is the gold standard for antibiotic susceptibility testing of *H. pylori*; the ADM results were therefore used as the reference for validating the E-test method. The samples were grouped into “sensitive” and “resistant” according to the EUCAST clinical breakpoint criteria [[1](#_ENREF_1)], and these nominal data were then used to evaluate the essential agreement between the two methods, defined as the samples where they both gave the same result. To check if the agreement occurred by chance, Cohen’s kappa analysis was performed, with the results interpreted as fair (0.21–0.40), moderate (0.41–0.60), substantial (0.61–0.80), and almost perfect (0.81–0.99). McNemar resistant proportion comparisons were calculated for the MIC results. The statistical analyses were performed using the SPSS statistical software package version 23.0 (IBM Corp., Armonk, NY, USA).

To better understand the agreement of the MIC results (interval data), we performed the analysis based on the CLSI’s recommendations for method comparisons. The agreement between the MIC results was analyzed by the non-parametric approach proposed by Bland and Altman with Krouwer modification because the gold standard was included in the analysis. The *y*-axis was the difference between each ADM measurement and the corresponding E-test measurement while the *x*-axis was the ADM values. The limits of agreement were 2.5 and 97.5 percentiles. [[20](#_3j2qqm3)[[2](#_ENREF_2)].

Scatter plot and Passing–Bablok analyses were performed using R environment ver. 3.5.1 with the mcr package [3[3](#_ENREF_3)]. The Passing–Bablok method involved plotting the MIC values obtained from the ADM and E-test against each other and finding the intercept and slope of the regression line. This method also calculated the confidence interval for the regression model to determine whether there was a significant difference between two methods. Receiver operating characteristic analysis was used to evaluate the sensitivity, specificity, and area under the curve (AUC) of the E-test results relative to those of the ADM. We also analyzed the major and very major error rate between the methods, defined as the discrepancy in categorical results between them (i.e., where one method gave a result of “sensitive” and the other of “resistant”). A very major error was defined as when a strain that was resistant according to ADM was assessed as “sensitive” by the E-test; this would result in the patient not receiving effective antibiotics, allowing the infection to continue. A major error was defined as when the strain was sensitive according to ADM but assessed as “resistant” by the E-test.

References

1. EUCAST D. Document EDEF. 3.1, June 2000: Determination of minimum inhibitory concentrations (MICs) of antibacterial agents by agar dilution. Clin Microbiol Infect. 2000;6:509-15.

2. Krouwer JS. Why Bland–Altman plots should use X, not (Y+ X)/2 when X is a reference method. Statistics in medicine. 2008;27(5):778-80.

3. Manuilova E, Schuetzenmeister A, Model F. mcr: method comparison regression. R package version 1.2. 1. 2014.

**Passing-Bablok Regression Results**


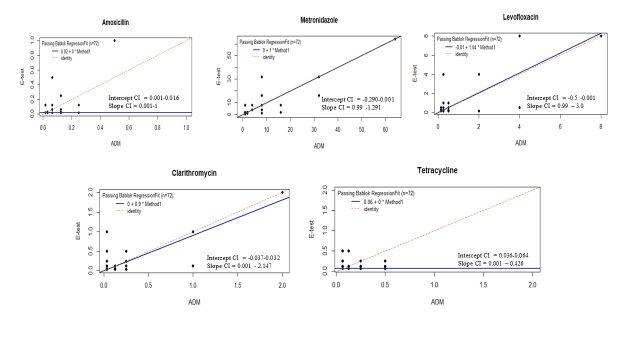


**Figure S1. Passing Bablok Regression of ADM and E-test**

Passing–Bablok regression plots. The regression formula is y + b * method 1 (E-test), thus y is corresponding to the value of intercept and b is corresponding to the value of the slope. The confidence interval of the slope and interval mentioned in the picture are used to draw the conclusion. The identity line is drawn by the formula x = y. The plots showed no significant differences in slope and intercept between the agar dilution method (ADM) and E-test for metronidazole, clarithromycin, and levofloxacin, but not for amoxicillin and tetracycline.
